# Review of the role of imaging in the diagnosis of priapism

**DOI:** 10.1038/s41443-024-00928-0

**Published:** 2024-06-11

**Authors:** Conrad von Stempel, Miles Walkden, Alex Kirkham

**Affiliations:** 1 Department of Radiology, University College London Hospital, London, UK; 2 Division of Surgery and Interventional Science, Faculty of Medicine, UCL, London, UK

**Keywords:** Sexual dysfunction, Diagnosis

## Abstract

Imaging has a specific role in the diagnosis and management of priapism. The primary imaging modality is ultrasound with colour Doppler (CDUS) which can accurately assess the hemodynamics of the cavernosal arteries. This is particularly useful in equivocal cases and can help differentiate ischemic from non-ischemic priapism as well as confirm the presence and location of arterio-venous fistulae post penile trauma. Furthermore, CDUS is invaluable in the post treatment follow up of non-ischemic priapism. Contrast enhanced magnetic resonance imaging (MRI) can demonstrate the extent of cavernosal necrosis in ischemic priapism and in conjunction with computer tomography (CT) has an important role in excluding underlying malignancy. MRI and CT angiography are used to evaluate pudendal arterial anatomy, which can be extremely variable and aids in the management of non-ischemic priapism. In selected cases of non-ischemic priapism, catheter angiography and transcatheter embolization of arteriovenous fistulae is an effective treatment. This review will examine the specific roles of different imaging modalities in the subtypes of priapism as well as highlight some of the pitfalls encountered in imaging.

## Introduction

Priapism is an uncommon diagnosis with an incidence of 1.5 per 100,000 person-years (95% confidence interval 0.2 to 2.8) [1].

The pathophysiology is divided broadly into two main groups based on the hemodynamic state. Ischemic priapism is by far the most common subtype (95% of cases) and is likened to a compartment syndrome of the penis; it is often idiopathic but can be associated with clotting dyscrasias, malignancy, sickle cell disease, and various medication; non-ischemic priapism is less frequent (<5% of all priapism cases) and results from arteriovenous fistula (AVF) formation within the corpora cavernosa secondary to perineal trauma [2]. The third group is stuttering priapism with short-lived episodes of abnormal painful tumescence that is self-limiting and seen almost exclusively in men with homozygous sickle cell disease [3].

Diagnosis of priapism primarily relies on clinical history and physical examination. Ischemic priapism presents acutely with pain, constituting a medical emergency, while non-ischemic priapism manifests as non-tender partial tumescence appearing days to weeks after the initial injury. The delay in presentation is believed to be as a result of normal physiological night-time erections leading to increased flow in the contused tissue and subsequent development of the AVF [4]. Intracorporal blood gas aspiration is the primary investigation in priapism and can accurately identify the characteristic low oxygen tension in ischemic priapism (PO_2_ typically less than 30 mmHg, PH < 7.2) and normal oxygen tensions in non-ischemic priapism [5].

Imaging is generally not required in the acute diagnosis of priapism and certainly, at least in ischemic priapism, imaging should not lead to a delay in initializing treatment. However, it does have an important role to play in the confirmation and treatment of non-ischemic priapism and in assessing the viability of the corpora in prolonged ischemic priapism. Imaging can be divided into (a) functional imaging with assessment of local cavernosal blood flow, (b) structural imaging to assess penile fibrosis and vascular anatomy, and (c) abdominal and pelvic imaging to identify a potential underlying cause.

## Imaging techniques

CDUS and MRI are the most commonly used imaging techniques to image the penile tissues in priapism, whilst CT angiography (CTA) and magnetic resonance angiography (MRA) are used to investigate underlying malignancy and plan arterial anatomy for embolization in cases of non-ischemic priapism [6, 7].

CDUS has the potential to be the most useful imaging modality. Ultrasound has excellent soft tissue resolution to accurately identify AVF [8], structural abnormalities such as hematoma, fibrosis, and tunical plaques [9], and with the addition of Doppler waveform sampling, helps determine the hemodynamic state of the penis [10].

CDUS should be performed in a standardized reproducible way and the authors recommend scanning the ventral crura at the position where the cavernosal arteries perforate the tunica [11]. Imaging of the cavernosal arteries in the midshaft and tips can be useful to identify common intrapenile arterial anatomical variants such as dorsal penile artery perforating branches, transverse root collaterals which connect the left and right cavernosal arteries, and accessory cavernosal arteries [12]. Insonation of the cavernosal arteries requires optimization of the scale and gate size for these small calibre vessels (1–3 mm) as well as correction of the angle of insonation to 30° and 60°. At least 3 cardiac cycles should be sampled to increase accuracy of the assessment [13].

MRI of the penis is commonly performed in the management of priapism with a combination of small field of view T2 weighted sequences in three orthogonal planes, larger field of view pelvic sequences, and dynamic post-gadolinium sequences. The small field of view anatomical T2 weighted sequences can demonstrate the extent of necrosis and fibrosis in ischemic priapism [14] and can identify lesions that may mimic true priapism such as metastatic infiltration [15] and segmental thrombosis [16, 17].

Post-contrast sequences are useful to estimate corporal tissue viability particularly in late presenting ischemic priapism [14] and also to help locate AVFs in non-ischemic priapism.

## Non-ischemic priapism

In suspected non-ischemic priapism, there is often a relevant history that includes high-impact falls astride, such as in motorcycle, cycling, skateboarding, and industrial building site accidents [18, 19]. There are occasional cases when a relatively indolent perineal injury is the cause and may be forgotten or ignored by the patient, this is especially when patients are embarrassed by their condition, in cases with delayed presentation, and in children [20]. Corporal aspiration in non-ischemic priapism is generally diagnostic (with high oxygen tension in intracavernosal blood) but imaging is required to confirm the presence and laterality of AVFs. CDUS can identify the site of cavernosal injury in over 70% of cases [21, 22]. AVFs are most often found in the crura at the site of contusion between the erectile tissues and the underling pubic bone and are bilateral in up to 20% of cases [18, 23].

**Fig. 1:**
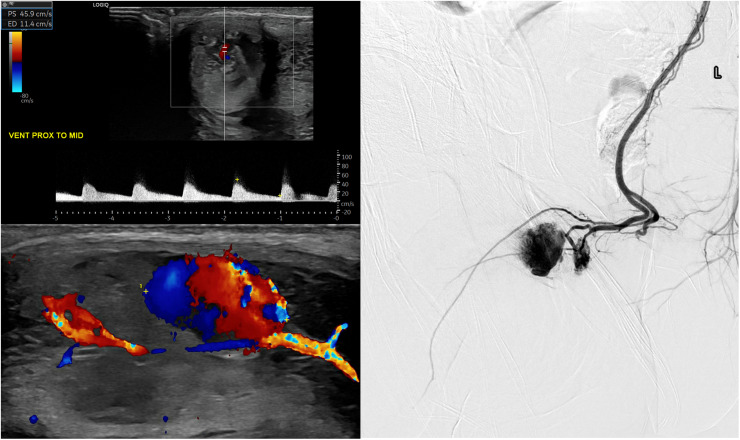
Top left – Right sided corporal cavernosal artery waveforms showing characteristic low resistance waveforms (peak systolic velocity 45.9 cm/s and end diastolic velocity of 11.4 cm/s). Bottom left – Large bilobed pseudoaneurysm arising from proximal right cavernosal artery as demonstrated by classical blue and red ‘ying yang’ appearance. Right – catheter angiogram from the right side of the pelvic, selective catheterisation of the right accessory pudendal artery (variant anatomy) that supplies the penis. Large bilobed fistula is demonstrated clearly by the blush of contrast.

The CDUS waveform seen in non-ischemic priapism is distinctive with a low resistance high-net-flow pattern. A peak systolic velocity (PSV) of well over 50 cm/s and end diastolic velocity (EDV) of over 10 cm/s with an accompanying resistive index (RI) of <0.7 is usually measured in the affected cavernosal artery [18]. The cavernosal artery waveforms in the contralateral corporal body are also often abnormal even in the absence of bilateral AVFs, due to the semipermeable nature of the midline septum and anastomotic arcades within the glans penis and perforating vessels. CDUS reports should note any accessory cavernosal arteries or variant anatomy because embolization of AVFs relies on excluding all the feeding vessels and early failure of embolization is commonly due to recanalization from collateral vessels [8] (see Fig. 1).

**Fig. 2:**
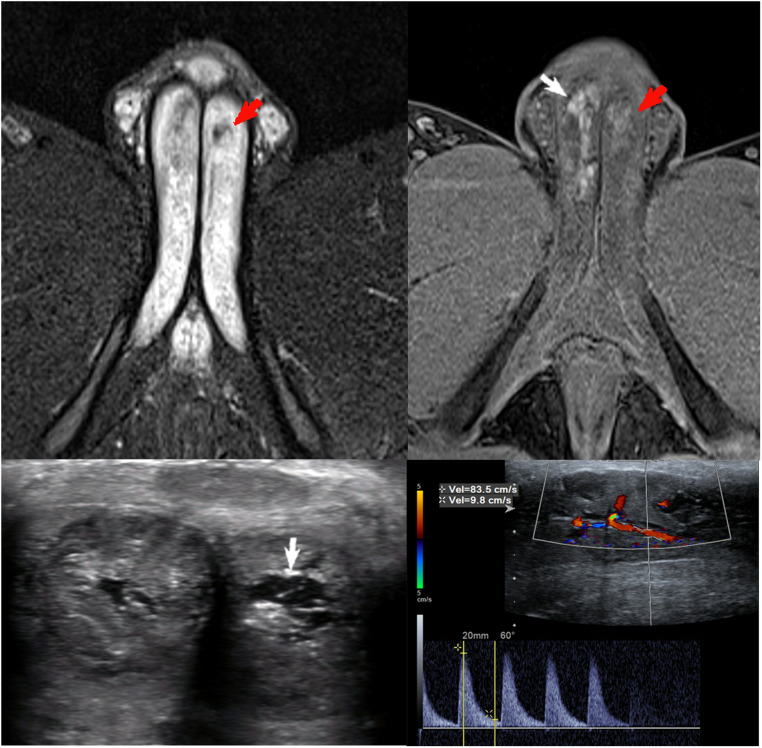
MRI and CDUS images in non-ischemic priapism. Top left – T2w MRI axial section through the crura/proximal corporal cavernosa showing ill defined low T2 signal in the central corpora (red arrow), this likely represents contused tissue with hematoma. Top right – T1w FS post contrast arterial phase axial section at the same position as the adjacent image. Arrow indicating the early enhancement and contrast blush at the site of arterio-venous fistula (red arrow). A similar contralateral blush is also seen indicating bilateral injury (white arrow). Bottom left – axial ultrasound image at the same level showing an ill-defined hypoechoic region in the central corpus cavernosum indicating the sac of the AVF (white arrow). Bottom right – longitudinal image of the left corpus cavernosum showing characteristic low resistance waveforms (PSV 83.5 cm/s, EDV 9.8 cm/s) indicative of an arteriovenous fistula.

Cross-sectional imaging with CTA or MRA can be useful to identify the laterality of the AVFs and to aid planning catheter angiography and embolization [6] (see Fig. 2). The presence of accessory pudendal arteries is seen in nearly 30% of cases [24]. Variant intrapenile arterial anatomy with connection of the two internal pudendal or common penile arteries (transverse root collaterals) is extremely common and seen in up to 90% in some series [12, 25, 26]. Appreciation of these variants before subselective angiography is crucial to the successful treatment of the fistula to avoid missing potential collateral pathways that would lead to recanalization of the AVF [8]. In the authors’ practice, a flush aortogram performed prior to cannulation of the pelvic vessels at the time of embolization normally identifies variant internal-iliac anatomy, potentially negating the need for pre-operative cross-sectional imaging. Cross-sectional angiography (CTA or MRA) is reserved for failed embolization attempts in order to identify variant supply to the penis. One of the most commonly missed variant supplies is an aberrant accessory pudendal artery arising from the obturator artery, when the obturator artery itself arises from the external iliac artery along with the inferior epigastric artery [27].

After resolution of non-ischemic priapism, either after conservative or embolization management, follow-up CDUS is recommended at 1–2 weeks to confirm occlusion of the AVF [8]. It is common to see a slow resolution of the CDUS waveforms back to a normal baseline for up to 6 months after resolution of the priapism. This is thought to be due to reactive hyperemia as part of the healing process and potentially delayed healing of small arteriovenous shunts that are not a distinct AVF [8, 18].

Erectile dysfunction post-treatment for non-ischemic priapism occurs in up to 39% of cases [18, 19, 23]. This can either be due to arteriogenic causes due to compromise of the main cavernosal blood supply or potentially due to persisting micro arteriovenous connections, as evidenced by persisting low resistance resting CDUS waveforms. This is thought to lead to a veno-occlusive failure erectile dysfunction [18].

The difference between these two causes is important as arteriogenic incompetence and persisting veno-occlusive dysfunction necessitate different management strategies. Stimulated CDUS can be performed to exclude arterial insufficiency and MRI is occasionally required to demonstrate any structural injury or fibrosis that may have occurred [28].

## Ischemic priapism

Ischemic priapism is generally diagnosed and managed without need for medical imaging in the initial setting. Imaging can be considered in cases where the diagnosis is in doubt (borderline gases) [29], in cases of prolonged priapism to assess corporal viability [14] and when evaluating the underlying cause of the priapism [7].

Evaluating CDUS waveforms in ischemic priapism can pose a diagnostic dilemma, especially in subacute and post-intervention scenarios, such as after phenylephrine injection or cavernosal shunt procedures [29].

**Fig. 3:**
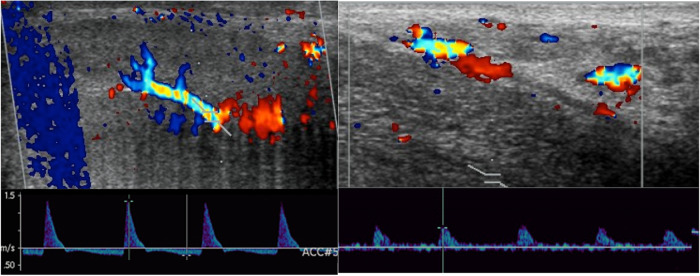
CDUS waveforms in different cases of ischemic priapism. Left image – waveforms in early ischemic priapism <24 h, shows a high resistance waveform with PSV 15 cm/s but reversal of flow in diastole EDV – 2 cm/s, indicative of very low net perfusion into the corpora. Right image – advanced >48 h ischemic priapism showing high resistance waveforms in the cavernosal artery – PSV < 5 cm/s EDV 0.

In cases of established ischemia that has persisted for several hours or days, there is typically minimal or no blood flow measurable in the corpora cavernosa. When flow is seen, it is characterized by a low-flow, high-resistance waveform, with PSV measuring less than 5 cm/s and EDV registering at zero [29]. In the early phase the waveform is much more akin to that seen in a normal erection with high-resistance waveforms, which is often accompanied by an elevated PSV (approximately 25 cm/s or higher), but notably, there is a reversal or absence of flow during diastole. This still signifies ischemia and results in a low mean blood flow in the penis. It can be misinterpreted as “high-flow” non-ischemic priapism if one solely interprets the PSV measurement without considering the overall waveform characteristics and net flow [29]. The different waveform patterns in ischemic priapism can be considered a spectrum of change, from the normal waveforms seen in an erection, to complete occlusion of the cavernosal arteries in established cavernosal necrosis (see Fig. 3).

Post cavernosal shunt surgery, CDUS waveforms vary greatly and are unlikely to be useful to predict clinical outcomes such as erectile function or need for implant surgery [29]. CDUS can have a role in excluding an iatrogenic AVF that may have occurred secondary to cavernosal arterial laceration during shunt surgery or corporal decompression [30].

**Fig. 4:**
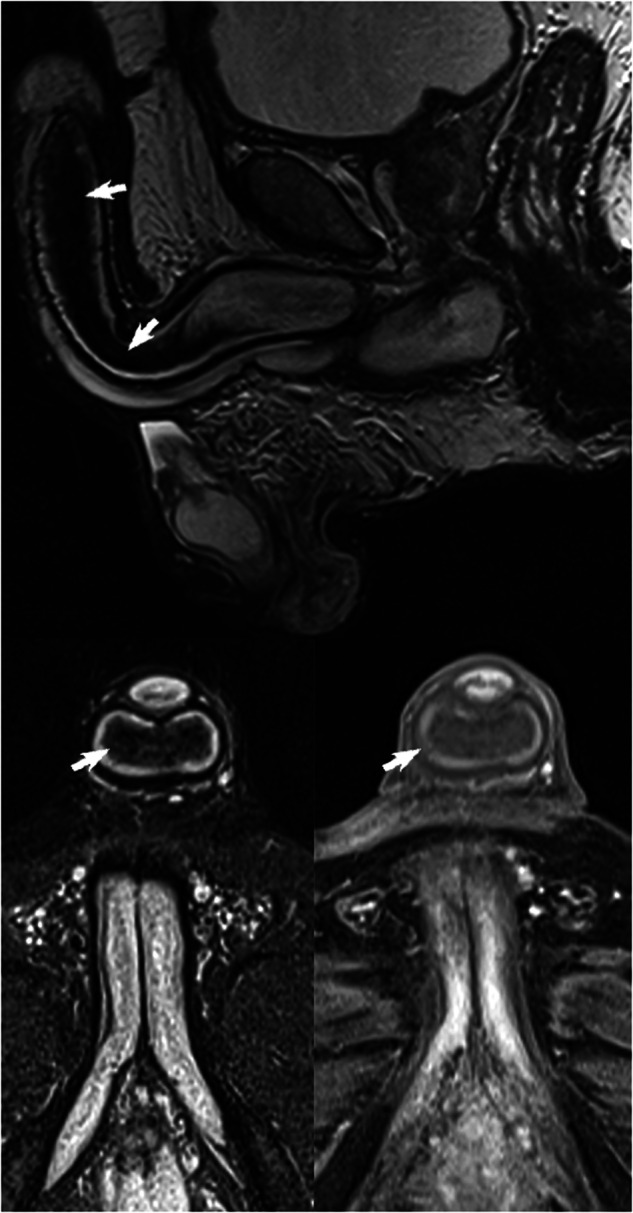
MRI in ischemic priapism. Top and bottom left image – T2 sagittal plane showing extensive low T2 signal in the central corpora cavernosa indicating necrotic/infarcted tissue. Bottom right image – T1FS post contrast at a similar level showing no central cavernosal enhancement. A thin rind of enhancement in the corpora with preserved enhancing crural tissue is seen. These appearances likely relate to dorsal penile artery branches perfusing the peripheral corporal tissue.

Contrast enhanced MRI of the penis is useful to evaluate the extent of established necrosis and fibrosis in the corpora (as demonstrated on T2 weighted sequences). Post-contrast sequences can give an indication of the amount of viable tissue that correlates well with corporal biopsy histology [14]. This is useful when making decisions between acute implant surgery and the potential for success of shunt surgery, particularly in ischemic priapism that has lasted for more than 72 h [14] (see Fig. 4).

**Fig. 5:**
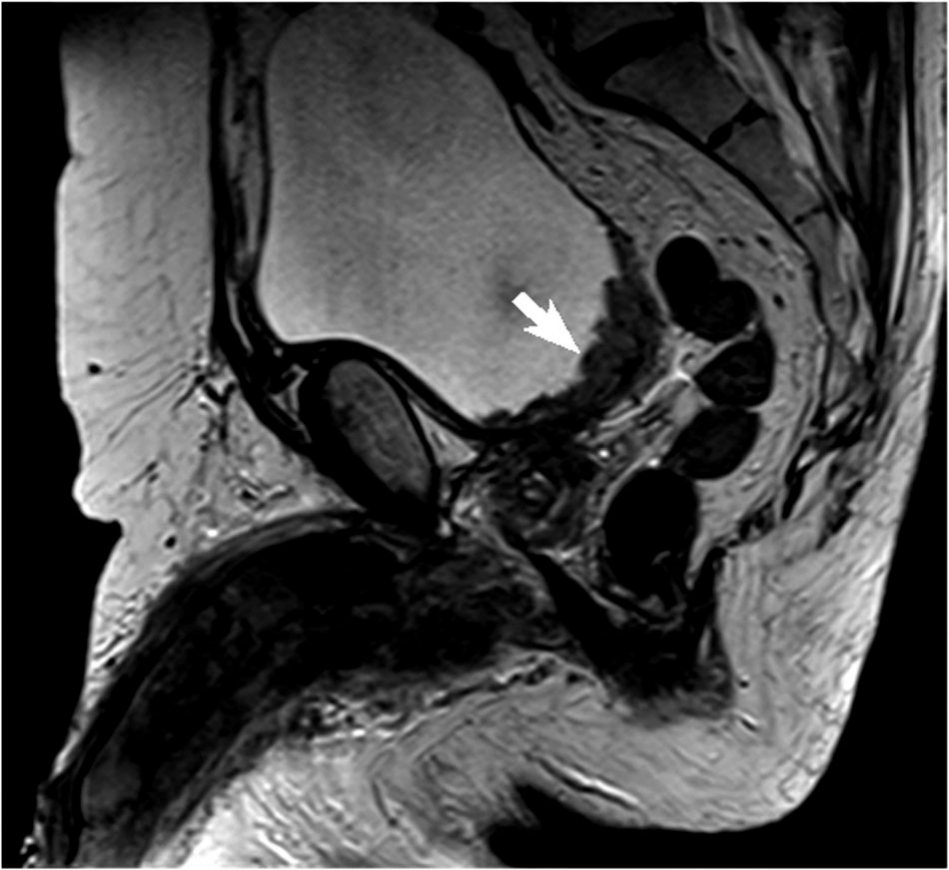
Sagittal plane T2w MRI pelvis showing a large plaque like bladder tumour (white arrow) that was confirmed transitional cell carcinoma. The abnormal low T2 tissue is seen extending through the bladder neck, prostate, and into the urethra and filling the penis with heterogenous low T2 tissue.

Malignant infiltration of the penile tissues is a rare subtype that can lead to either ischemic priapism through clotting dyscrasias, such as seen in hematological malignancy or a priapism like state with penile metastases infiltrating the corpora [31]. In one series of over 400 men with ischemic priapism, an underlying malignant cause was found in 11 cases (<3%), most frequently local invasion from bladder and prostate cancer. The authors recommend further investigation for occult malignancy in men with ischemic priapism with no clear precipitating cause, particularly in those with anemia, although penile metastases without a known primary are very rare [7]. Imaging is usually performed with a CT chest, abdomen, and pelvis and MRI pelvis as part of the MRI penis protocol (see Fig. 5).

## Stuttering priapism

Stuttering priapism is poorly understood and is characterized by self-limiting periods of painful erections similar to ischemic priapism. Ischemic priapism in men with sickle cell disease is common due to sickle crisis with an incidence of 35%. Furthermore, up to 72% of these men had associated stuttering priapism [3]. Although imaging does not have a major role in the diagnosis of this condition as the priapism, CDUS in men with a history of stuttering priapism reveals an interesting finding. In the flaccid state, the CDUS waveforms show an abnormal low resistance waveform with raised and labile diastolic flows. This is thought to represent an imbalance between the vasodilatory and vasoconstrictive mechanisms in the corpora as a result of sickle cell ischemic crises damaging the normal regulatory mechanisms in the cavernosal endothelium [32]. This finding of a low resistance waveform should not be misinterpreted as a development of non-ischemic priapism or an AVF in men with a relevant prior history of ischemic priapism and sickle cell disease.

## Conclusion

CDUS of the penis is practically the most useful imaging test in priapism. It should be performed principally to measure the altered hemodynamics and confirm the diagnosis when there is diagnostic doubt between ischemic and non-ischemic priapism. Furthermore, unstimulated CDUS should be performed routinely in the follow up of non-ischemic priapism. There are pitfalls in CDUS interpretation, particular if PSV is considered in isolation without considering the entire waveform, after shunt surgery, and in cases of stuttering priapism. CTA and MRA can help plan AVF embolization and identification of variant arterial anatomy in non-ischemic priapism. MRI has an important role in excluding penile metastasis in malignant infiltration mimicking ischemic priapism, as well as assessing the extent of corporal necrosis and potentially the viability of erectile tissue in late presenting ischemic priapism.

## References

[1] Eland IA, van der Lei J, Stricker BHC, Sturkenboom MJCM. Incidence of priapism in the general population. Urology. 2001;57:970–2. https://www.sciencedirect.com/science/article/pii/S0090429501009414. 11337305 10.1016/s0090-4295(01)00941-4

[2] Kuefer R, Bartsch G, Herkommer K, Krämer SC, Kleinschmidt K, Volkmer BG. Changing diagnostic and therapeutic concepts in high-flow priapism. Int J Impot Res. 2005;17:109–13. 15229624 10.1038/sj.ijir.3901257

[3] Adeyoju AB, Olujohungbe ABK, Morris J, Yardumian A, Bareford D, Akenova A, et al. Priapism in sickle-cell disease; incidence, risk factors and complications - an international multicentre study. BJU Int. 2002;90:898–902. 12460353 10.1046/j.1464-410x.2002.03022.x

[4] Wu A, Lue T. Commentary on high flow, non-ischemic, priapism. Transl Androl Urol. 2012;1:109–12. www.amepc.org/tau. 26816695 10.3978/j.issn.2223-4683.2012.06.04 PMC4708203

[5] Bivalacqua TJ, Allen BK, Brock G, Broderick GA, Kohler TS, Mulhall JP, et al. Acute ischemic priapism: an AUA/SMSNA guideline. J Urol. 2021;206:1114–21. 34495686 10.1097/JU.0000000000002236

[6] White C, Gulati M, Gomes A, Rajfer J, Raman S. Pre-embolization evaluation of high-flow priapism: Magnetic resonance angiography of the penis. Abdom Imaging. 2013;38:588–97. 22923172 10.1007/s00261-012-9936-9

[7] James Johnson M, Hallerstrom M, Alnajjar HM, Frederick Johnson T, Skrodzka M, Chiriaco G, et al. Which patients with ischaemic priapism require further investigation for malignancy? Int J Impot Res. 2020;32:195–200. 30996267 10.1038/s41443-019-0141-z

[8] Bertolotto M, Quaia E, Mucelli FP, Ciampalini S, Forgées B, Gattuccio I. Color Doppler imaging of posttraumatic priapism before and after selective embolization. Radiographics. 2003;23:495–503. 12640162 10.1148/rg.232025077

[9] Punjani N, Stern N, Brock G. Characterization of septal and punctate scarring in Peyronie’s disease. Urology. 2018;118:87–91. 29800632 10.1016/j.urology.2018.05.014

[10] Chiou RK, Aggarwal H, Chiou CR, Broughton F, Liu S. Colour Doppler ultrasound hemodynamic characteristics of patients with priapism before and after therapeutic interventions. Can Urol Assoc J. 2009;3:304–11. http://www.ncbi.nlm.nih.gov/pubmed/19672444. 19672444 10.5489/cuaj.1125 PMC2723895

[11] Kim S, Paick J, Lee S, Choi B, Yeon K, Han M. Doppler sonography of deep cavernosal artery of the penis: variation of peak systolic velocity according to sampling location. J Ultrasound in Med. 1994;13:591–4. 7933026 10.7863/jum.1994.13.8.591

[12] Bookstein J, Lang E. Penile magnification pharmacoarteriography: details of intrapenile arterial anatomy. Am J Roentgenol. 1987;148:883–8. 3107359 10.2214/ajr.148.5.883

[13] Hoyt K, Hester FA, Bell RL, Lockhart ME, Robbin ML. Accuracy of volumetric flow rate measurements: an in vitro study using modern ultrasound scanners. J Ultrasound Med. 2009;28:1511–8. 19854966 10.7863/jum.2009.28.11.1511 PMC3415042

[14] Ralph DJ, Borley NC, Allen C, Kirkham A, Freeman A, Minhas S, et al. The use of high-resolution magnetic resonance imaging in the management of patients presenting with priapism. BJU Int. 2010;106:1714–8. 20438564 10.1111/j.1464-410X.2010.09368.x

[15] Kendi T, Batislam E, Basar MM, Yilmaz E, Altinok D, Basar H. Magnetic Resonance Imaging (MRI) in penile metastases of extragenitourinary cancers. Int Urol Nephrol. 2006;38:105–9. 10.1007/s11255-005-0256-7. 16502062 10.1007/s11255-005-0256-7

[16] Asbach P, Oelrich B, Haase O, Lenk SV, Loening SA. Acute partial segmental thrombosis of the corpus cavernosum: imaging findings on ultrasound, computed tomography, and magnetic resonance imaging. Clin Imaging. 2008;32:400–2. 18760731 10.1016/j.clinimag.2008.02.022

[17] Koller C, Fustok J, Hua J, Triche B, Hellstrom W, Khera M, et al. Unilateral corporal cavernosum partial thrombosis: a challenging presentation and management. Urology. 2021;152:12–4. 33600835 10.1016/j.urology.2021.02.009

[18] von Stempel C, Shazhad R, Walkden M, Castiglione F, Muneer A, Ralph D, et al. Therapeutic outcomes and analysis of Doppler findings in 25 patients with non-ischemic priapism. Int J Impot Res. 2024;36:55–61. 10.1038/s41443-023-00719-z PMC10810751 37311966

[19] Ciampalini S, Savoca G, Buttazzi L, Gattuccio I, Pozzi Mucelli F, Bertolotto M, et al. High-flow priapism: treatment and long-term follow up. Urology. 2002;59:110–3. 11796291 10.1016/s0090-4295(01)01464-9

[20] Aja Obasi A, Sunday Omebe WE. Prolonged ischemic priapism in an adolescent with sickle cell anemia: challenges of management. J Case Rep Images Surg. 2024;10:15–8.

[21] Hakim LS, Kulaksizoglu H, Mulligan R, Greenfield A, Goldstein I. Evolving concepts in the diagnosis and treatment of arterial high flow priapism. J Urol. 1996;155:541–8. https://www.sciencedirect.com/science/article/pii/S0022534701664449. 8558656

[22] Bertolotto M, Zappetti R, Pizzolato R, Liguori G. Color Doppler appearance of penile cavernosal-spongiosal communications in patients with high-flow priapism. Acta Radiol. 2008;49:710–4. https://www.tandfonline.com/doi/abs/10.1080/02841850802027026. 18568565 10.1080/02841850802027026

[23] Savoca G, Pietropaolo F, Scieri F, Bertolotto M, Mucelli FP, Belgrano E. Sexual function after highly selective embolization of cavernous artery in patients with high flow priapism: long-term followup. J Urol. 2004;172:644–7. 15247752 10.1097/01.ju.0000132494.44596.33

[24] Breza J, Aboseif SR, Orvis BR, Lue TF, Tanagho EA. Detailed anatomy of penile neurovascular structures: surgical significance. J Urol. 1989;141:437–43. 2913372 10.1016/s0022-5347(17)40789-0

[25] Bahren W, Gall H, Scherb W, Stief C, Thon W. Arterial anatomy and arteriographic diagnosis of arteriogenic impotence. Cardiovasc Interv Radiol. 1988;11:195–210. 10.1007/BF02577004 3147134

[26] Curet P, Grellet J, Perrin D, Bousquet JC, Jardin A. Technical and anatomic factors in filling of distal portion of internal pudendal artery during arteriography. Urology. 1987;29:333–8. 3824738 10.1016/0090-4295(87)90087-2

[27] Henry BM, Pękala PA, Vikse J, Sanna B, Skinningsrud B, Saganiak K, et al. Variations in the arterial blood supply to the penis and the accessory pudendal artery: a meta-analysis and review of implications in radical prostatectomy. J Urol. 2017;198:345–53. 28202357 10.1016/j.juro.2017.01.080

[28] Zacharakis E, Ralph DJ, Walkden M, Muneer A. Distal corpus cavernosum fibrosis and erectile dysfunction secondary to non-ischaemic priapism. Arch Ital Urol Androl. 2015;87:258–9. 26428655 10.4081/aiua.2015.3.258

[29] von Stempel C, Zacharakis E, Allen C, Ramachandran N, Walkden M, Minhas S, et al. Mean velocity and peak systolic velocity can help determine ischaemic and non-ischaemic priapism. Clin Radiol. 2017;72:611.e9–611.e16. 10.1016/j.crad.2017.02.021 28351471

[30] Lutz A, Lacour S, Hellstrom W. Conversion of low-flow to high-flow priapism: a case report and review (CME). J Sexual Med. 2012;9:951–4. 10.1111/j.1743-6109.2012.02692.x 22462585

[31] Da Silva Gaspar SR, Nunes A, Dias JS, Lopes T. Malignant priapism: penile metastasis originating on a primary prostate adenocarcinoma. Urol Ann. 2015;7:391–5. 26229335 10.4103/0974-7796.152030 PMC4518384

[32] Patel U, Sujenthiran A, Watkin N. Penile Doppler ultrasound in men with stuttering priapism and sickle cell disease-a labile baseline diastolic velocity is a characteristic finding. J Sexual Med. 2015;12:549–56. 10.1111/jsm.12756 25424427

